# Public health. Social context and action

**Published:** 2012-04-27

**Authors:** Thomas E Dorner

**Affiliations:** Institute of Social Medicine, Centre for Public Health, Medical University Vienna, Rooseveltplatz 3, 1090 Wien E-mail: thomas.dorner@meduniwien.ac.at

Health is determined by the conditions in which we live our everyday lives. Those conditions include the social, cultural, economic, educational, and occupational as well as the physical and mental environment, which influence health behaviour and lifestyle and so the health status. Disparities in those determinants between social groups lead to inequalities in health. Those issues are contents of the book ‘Public Health. Social context and action.’ Knowledge of such social determinants of health, both, on individual level as well as on the level of communities is crucial to health planning. Thus, the contents of this book are of great importance for persons who are engaged and interested in integrated care issues.

The book is written by 23 different authors, mainly from the UK. The book consists of two introductory chapters and after that of two main parts, each part with eight sub-chapters. Thus, the book comprises 18 separately authored chapters, part 1 is titled as ‘the social context of public health’, and part 2 as ‘public health action’.

The first chapter of the book gives an outlook on the contents of the two main parts of the book and summarizes each chapter. Additionally, the main messages and conclusions of the chapters that will follow are highlighted in a key deduction section. In the second introductory chapter a historical overview of public health in the UK is given. In some points reference is made to the US or Europe. The chapter also provides the readers with an overview of the current public health agenda in the UK.

The first main part of the book, which comprises chapters 3–10, gives a theoretical overview of the impact of socio-economic variables on individual and population health. In these chapters a comprehensive diversity of potential influencing factors on the health status is presented. In chapter 3 the socio-political context of public health is reflected. Demographic aspects like mortality, fertility, migration and trends for these demographic patterns in Great Britain are presented and their public health impacts, such as changes in disease frequency (epidemiological transition), are discussed. In the next chapter a model for the interactions between social determinants, health behaviour, and health inequalities is presented and the consequences for health policies discussed. The association between socio-economic variables and health is illustrated by figures of life expectancy by occupational class in England and Wales. The following chapter deals with the relationship between job characteristics and health. In this chapter environmental, psycho-social and gender aspects as well as resilience associated with work and which predict health status are analysed. Trends for the occupational composition in the labour force are shown. Chapter 6 addresses social capital and social exclusion as resources or obstacles for health and well-being. Social capital here is operationalized by neighbourhood attachment, the social network, and civic participation scores. In a large British database the association between social capital and happiness and satisfaction with life is presented. The role of ethnicity and racism in health disparities are topics of the following chapter. Ethnic factors, which should be more regarded as cultural or group-based identity aspects predict health status through health behaviour, racial discrimination, and access to health services. Patterns of ethnicity and health are presented by means of analysing data from an English health survey. Chapter 8 focusses on disability as an inequality issue regarding education, work, healthcare, housing, social care, leisure and politics, all predictors and determinants of health. According to the authors, disabled people represent a special target group for empowerment, health promotion and public health.

Mass media, e.g. television, film, print media, and the internet, as well as traditional public health campaign materials used by practitioners often transport health information, mainly focussing on promoting a more healthy lifestyle. In chapter 9 these phenomena and their impact on the health status are discussed on the example of case studies with mass media public health campaigns carried out in the UK. The last chapter of the first part of the book focusses on globalization and public health policy. The role of globalization and the role of international organizations such as the World Health Organization and the United Nations in communicable diseases like HIV/AIDS or tuberculosis, and in smoking are highlighted. In those areas globalization is creating challenges but also opportunities.

The second part of the book, which comprises of chapters 11ߝ18, focusses on public health action and hence, on the translation of theory into practical application. In this part strategic public health initiatives and projects in the UK are discussed especially with regard to their effectiveness and efficacy. The second part of the book begins with a discussion about terms and concepts of healthy public policies and if those policies are merely theory or reality in the UK. A discussion about multi-disciplinarity and multi-agency partnerships of public health action follows. A description and discussion of UK public health programmes follow which focus on the reduction of health inequalities in the UK, or setting-based programs like programmes at workplaces or in healthy cities, programmes to reduce the smoking-related health burden in the UK. The final chapter covers a project, where housing and the health and wellbeing aspects associated with that are highlighted.

One strength of the book is that in the first part, a comprehensive set of social determinants of health is presented, discussed, and illustrated with empirical data. The latter is, however, also part of a major limitation of the book, the presented data are mainly from, and the entire book focuses on aspects specifically in the UK. While non-British readers can also profit from the first part of the book, the second part is difficult to follow for readers who are not familiar with the British health care system in detail. Although the authors state in the introductory chapters that authors of the individual chapters are ‘drawing on international examples and comparisons where applicable and appropriate’, this is hardly the case in actuality, and the entire book is unnecessarily narrowly focussed on the UK. Concerning its relevance for integrated care, the book does discuss important aspects which also influence integrated care planning and organisation, but does not specifically allude to the subject. Overall, it is a book worth reading for those who want to improve health promotion in the UK, and it has a value for persons who want to get familiar with social determinants of health.

